# Field Trials and Baking Studies of Ultra‐Low Asparagine, Genome Edited (CRISPR/Cas9) and Mutant (TILLING) Wheat

**DOI:** 10.1111/pbi.70661

**Published:** 2026-04-01

**Authors:** Navneet Kaur, Sarah Raffan, Suzanne J. Clark, Shpresa Musa, Katharina Scherf, J. Stephen Elmore, Tanya Y. Curtis, Emma Honan, Nigel G. Halford

**Affiliations:** ^1^ Rothamsted Research Harpenden UK; ^2^ Department of Bioactive and Functional Food Chemistry, Institute of Applied Biosciences Karlsruhe Institute of Technology (KIT) Karlsruhe Germany; ^3^ Leibniz Institute for Food Systems Biology at the Technical University of Munich Freising Germany; ^4^ Professorship of Food Biopolymer Systems, TUM School of Life Sciences Technical University of Munich Freising Germany; ^5^ Department of Food and Nutritional Sciences University of Reading Reading UK; ^6^ Curtis Analytics Limited Harpenden UK

**Keywords:** acrylamide, asparagine synthetase, baking, CRISPR/Cas9, food safety, TILLING, wheat

## Abstract

Field trials were conducted of wheat ( *Triticum aestivum* ) cv. Cadenza in which asparagine synthetase gene, *TaASN2*, had been knocked out, either on its own or together with a partial knockout of the related gene, *TaASN1*, using CRISPR/Cas9. Chemical mutagenesis (TILLING) *TaASN2* nulls in the Claire background were also included. The main aim was to assess the free asparagine content of the grain and the conversion of free asparagine to acrylamide, a toxic contaminant, in bread, toast and biscuits. Over 2 years of trials combined, the *TaASN2* and *TaASN1/2* CRISPR knockouts resulted in a reduction of free asparagine in the grain of 59% and 93%, respectively, compared with Cadenza. The reduction in the *TaASN2* total knockout TILLING line compared with Claire was 50%. Yield was not affected in the edited lines but was reduced in the TILLING lines. Acrylamide in bread made from a *TaASN1/2* CRISPR line was below detection levels, while in a *TaASN2* CRISPR line it was 14% of the Cadenza control. Even after 4 min of toasting, acrylamide levels remained at 8% and 23%, respectively, of the control. The concentration in bread made from the TILLING *TaASN2* knockout was 21% that for the Claire control, rising to 46% after 4 min of toasting. Acrylamide in biscuits made from a *TaASN1/2* CRISPR line was reduced by 93% compared with the control. The relationship between acrylamide and colour was altered in the edited and mutant lines compared with the controls, with less acrylamide forming for the same degree of colour.

## 1 Introduction

Baking of wheat‐based doughs imparts texture, colour, flavour and aroma, and these positive factors are enhanced by toasting (exposure to a grill or other source of radiant heat). Many of the compounds that are involved are produced in the Maillard reaction, a complex series of reactions involving free amino acids and reducing sugars. However, not all products of the Maillard reaction are beneficial, with the processing contaminant, acrylamide (C_3_H_5_NO), which is derived from asparagine (Mottram et al. 2002; Stadler et al. 2002), probably the most undesirable (Kaur and Halford 2023). Acrylamide was discovered in common foods in 2002 (Tareke et al. 2002) and is present in foods derived from grains, beans, tubers and storage roots, primarily those that have been heated to > 120°C; in other words, those that have been baked, fried, roasted or toasted. It is classified as a Group 2a carcinogen (probably carcinogenic to humans) by the International Agency for Research on Cancer (IARC 1994). The margins of exposure to acrylamide in the diet with respect to its neoplastic effects have been described as a ‘concern’ by the European Food Safety Authority (EFSA)'s Expert Panel on Contaminants in the Food Chain (CONTAM) (CONTAM Panel 2015).

The European Union has gone further than any other regulatory body in imposing risk management measures for the presence of acrylamide in food and Commission Regulation (EU) 2017/2158 set Benchmark Levels for different food products. Current Benchmark Levels for wheat and other cereal products are 50 μg kg^−1^ for soft bread, 350 μg kg^−1^ for biscuits, crispbreads and rusks, and 300 μg kg^−1^ for breakfast cereals (European Commission 2017). The European Commission is understood to be preparing to present a new regulation setting out Maximum Levels and revised Benchmark Levels to the European Parliament (Kaur and Halford 2023). It would, of course, be illegal to sell a product that was found to contain more than the Maximum Level of acrylamide.

Food businesses face the challenge of modifying their processes to comply with these regulations while retaining the colour, flavour and aroma characteristics that define their products and are demanded by consumers. They are also vulnerable to blips in acrylamide levels caused by inconsistency in the acrylamide‐forming potential of the raw material that they use. For wheat and other cereal grains, free asparagine concentration is the determining factor for acrylamide formation (reviewed by Kaur and Halford 2023) and this is highly sensitive to crop management and environmental factors. Notably, sulphur deficiency causes a massive accumulation of free asparagine in wheat grain and must be avoided (Muttucumaru et al. 2006; Granvogl et al. 2007; Curtis et al. 2009, 2018; Oddy, Addy, et al. 2023). Nitrogen has the opposite effect (Claus et al. 2006; Martinek et al. 2009; Oddy, Addy, et al. 2023), and while the application of nitrogen fertiliser is essential for optimum yield and grain quality, we recommend that it should be accompanied with sulphur at a ratio of 10 N:1 S (Oddy, Addy, et al. 2023). Good disease control is also essential (Martinek et al. 2009; Curtis et al. 2016), with the signalling and response systems underlying the increase in free asparagine concentration induced by pathogen infection being one of the most fascinating and poorly understood aspects of plant/pathogen interactions (Perochon et al. 2019).

While these crop management factors are important, wheat that is produced under optimum crop management regimes still contains relatively high concentrations of free asparagine, and the concentration can vary from one location to another and from season to season (Curtis et al. 2009, 2018). This makes it difficult for food businesses to predict the acrylamide‐forming potential of the wheat grain they are using, and therefore the mitigation measures they need to apply in processing. The development of wheat varieties with reduced and more consistent concentrations of free asparagine in the grain would make regulatory compliance much easier for food businesses and potentially reduce the exposure of consumers to dietary acrylamide.

Asparagine is produced through the transfer of an amino group from glutamine to aspartate, generating asparagine and glutamate (Gaufichon et al. 2010; Xu et al. 2018). This reaction is catalysed by asparagine synthetase, and bread wheat ( *Triticum aestivum* ) has five asparagine synthetase genes in each of its three genomes, A, B and D (Xu et al. 2018). These are *TaASN1*, *TaASN2*, *TaASN3.1*, *TaASN3.2* and *TaASN4*, with *TaASN2* the most highly expressed in the grain, followed by *TaASN1* (Gao et al. 2016; Xu et al. 2018; Curtis et al. 2019). We have previously reported the development of wheat lines in which *TaASN2* was knocked out using CRISPR/Cas9 (Raffan et al. 2021, 2023). A field trial of an A genome null for *TaASN2* (Line 178), and total nulls for *TaASN2* (Lines 23.60 and 23.75) was carried out in 2021–2022 (Raffan et al. 2023; Kaur et al. 2024). Also included were four AB genome nulls, referred to as TILLING lines, derived from a selected line of a mutant population produced by ethyl methanesulphonate treatment of wheat cv. Cadenza seeds (Rakszegi et al. 2010). The mutated *TaASN2‐A2* gene from this line had been backcrossed into the cv. Claire background, which was chosen because Claire is a soft, biscuit‐type wheat that already lacks a B genome *TaASN2* gene due to a natural deletion (Oddy et al. 2021). The CRISPR lines showed a significant reduction of free asparagine concentration in the grain compared with the Cadenza control, by just over 50% in the total nulls and 14% in the A genome null (Raffan et al. 2023). The TILLING lines showed more variable responses when compared to Claire; however, when compared with a TILLING control that had come through the mutagenesis process but did not carry mutations in *TaASN2*, they did show significant reductions of 20%–40%, comparable with the reduction of 28% seen previously in A genome TILLING nulls in the Cadenza background and the tetraploid Kronos background (Alarcón‐Reverte et al. 2022).

Here we report the results of 2 years of field trials of the *TaASN2* knockout CRISPR wheat lines, alongside, for the first time, *TaASN2* total knockout/*TaASN1* partial knockout CRISPR lines and a *TaASN2* total null TILLING line. We describe acrylamide formation in bread made from the CRISPR and TILLING lines, with and without toasting, and in biscuits. Wild type Cadenza and Claire were used as controls (we consider these to be more relevant controls than null segregants and have shown previously that null segregants generated in the editing process had similar levels of free asparagine to wild type; Raffan et al. 2021). We discuss the results in the context of rapidly changing regulations on the presence of acrylamide in food and the commercial use of genome edited plants in the United Kingdom, European Union and elsewhere.

## 2 Results

Characterisation of *TaASN1/TaASN2* knockouts produced by CRISPR/Cas9 editing, and production of a total null for *TaASN2* by stacking of ethyl methanesulphonate (EMS)‐induced mutations in the Claire background.

The CRISPR editing of wheat ( *T. aestivum* ) cv. Cadenza reported previously (Raffan et al. 2021) was targeted at the *TaASN2* gene, which is the most highly expressed asparagine synthetase gene in wheat grain from mid‐development onwards (Gao et al. 2016). However, prior to the first field trials of the edited and TILLING lines, a second total *TaASN2* null, Line 59, was found to be edited in the closely related gene, *TaASN1* (Kaur et al. 2024). Four gRNAs had been used in the editing process (Raffan et al. 2021), with gRNA1 having seven mismatches with *TaASN1*, gRNA2 three mismatches, gRNA3 two mismatches and gRNA4 eight mismatches (Kaur et al. 2024). Protospacer adjacent motif (PAM) sites were present at the binding sites for gRNAs 1–3 but not gRNA4. Nucleotide sequence analysis revealed a 36 bp deletion in the B genome gene (*TaASN‐B1*) and a single nucleotide (T) insertion in the A genome gene (*TaASN‐A1*) adjacent to the gRNA3 site (Appendix S1). The D genome gene (*TaASN‐D1*) was not edited, meaning that Line 59 is a total null for *TaASN2* and a partial null for *TaASN1*. Wheat has three other asparagine synthetase genes: *TaASN3.1*, *TaASN3.2* and *TaASN4* (Gao et al. 2016; Xu et al. 2018). However, none of these has a PAM sequence at the gRNA binding sites (Kaur et al. 2024).

The TILLING lines were generated using mutations identified in a mutant population produced by EMS treatment of wheat cv. Cadenza seeds (Rakszegi et al. 2010). A mutation in the A genome *TaASN2* gene (*TaASN‐A2*) was identified in Line 1048 (EnsemblPlants reference Cadenza1048.chr3A.47849736 SNP). It comprises a G to A mutation at position 585 in the coding sequence with respect to the ATG start codon, with TGG becoming TGA (stop). The mutant gene encodes a protein of 194 amino acids. A mutation in the D genome *TaASN2* gene (*TaASN‐D2*) was identified in Line 0053. The D genome gene in EnsemblPlants has the reference TraesCS3D02G077300. Line 0053 is not listed in EnsemblPlants as one of those with a *TaASN‐D2* variant, but it carries a C to T mutation at position 385 of the coding region, changing a CGA codon to TGA (stop). The mutant gene encodes a 128 amino acid truncated protein. These mutations were stacked in the cv. Claire background by RAGT UK. Claire is a soft, biscuit wheat, and was chosen because it carries a natural deletion of the B genome *TaASN2* gene (*TaASN‐B2*) (Oddy et al. 2021).

We have previously reported a field trial including A and B genome TILLING nulls for *TaASN2* (Raffan et al. 2023). Two of these, TILLING 1AB and TILLING 2AB, were included in this study, but, for the first time, a total *TaASN2* null TILLING line, TILLING ABD, was also available. These lines contain the EMS‐generated mutations in the *TaASN‐A2* and (for TILLING ABD) *TaASN‐D2* genes described above, as well as the natural deletion of the *TaASN‐B2* gene (Oddy et al. 2021).

### 2.1 Field Trials

Field trials were carried out in 2022–2023 (trial 2318) and 2023–2024 (trial 2418). There were 56 × (6 m × 1.8 m) randomised plots (Appendix S2), including five of each of edited lines 23.60 and 23.75 (*TaASN2* total knockouts), 178.35 (A genome *TaASN2* knockout), lines 59.26 and 59.84 (*TaASN2* total knockouts/*TaASN1* partial knockout), and TILLING lines 1AB, 2AB (A and B genome *TaASN2* nulls) and ABD (total *TaASN2* null). There were eight plots of each of the Cadenza and Claire controls.

### 2.2 Yield and Thousand Grain Weight

**FIGURE 1:**
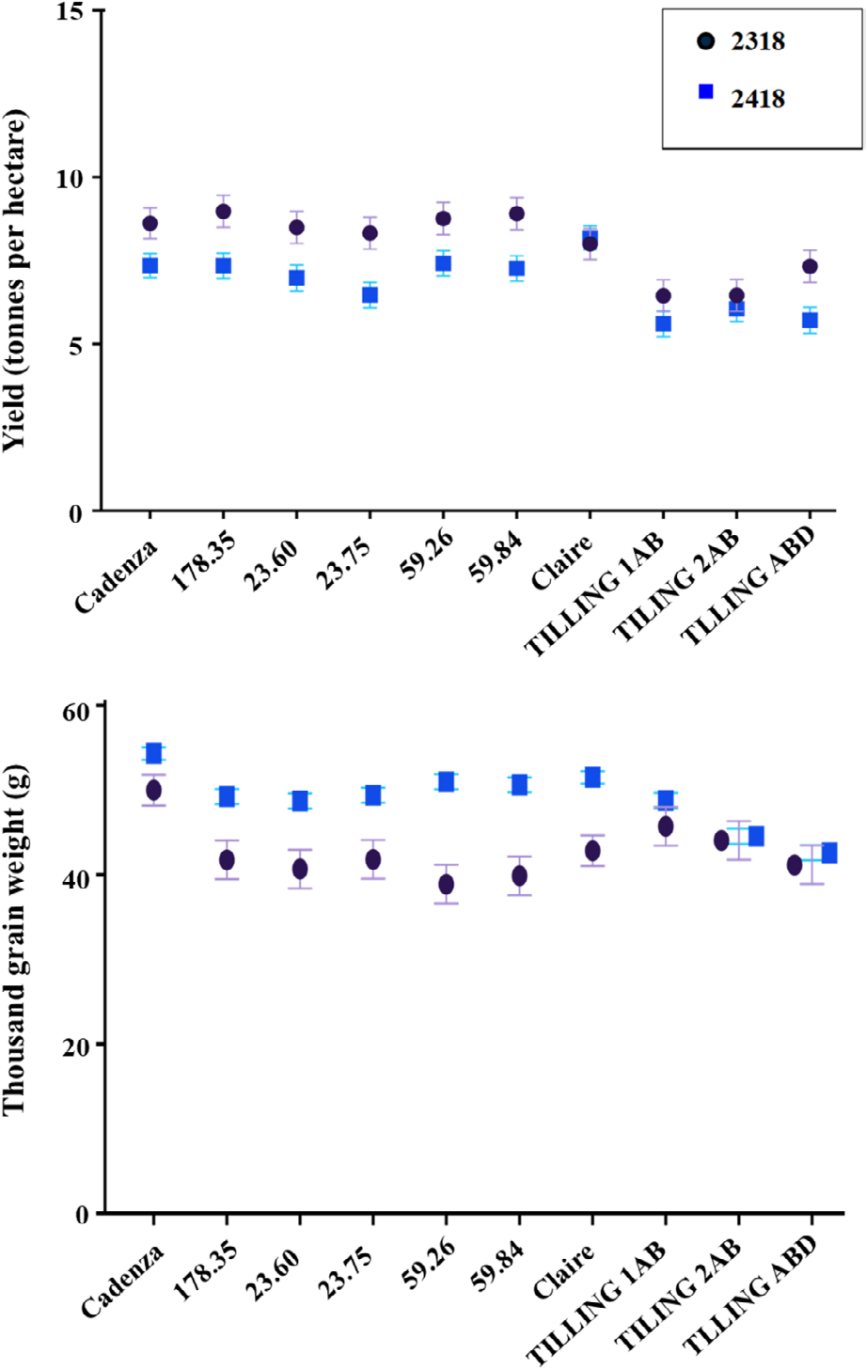
Yield (upper panel) and thousand grain weight (lower panel) of different lines of wheat grown in the 2318 and 2418 field trials, as indicated. The mean and standard error of the mean are shown in each case.

Yield and thousand grain weight (TGW) data from both field trials were combined across trials using a linear mixed model (LMM) meta‐analysis with a two‐way fixed model (trial × genotype), allowing a separate random model for each trial, including separate estimates of residual variance. In all analyses, nested contrasts were incorporated to compare between and within groups of genotypes. The yield data and statistical analysis are given in Appendix S3 and shown graphically in Figure 1, upper panel. Overall, yield was slightly lower in 2418 than 2318.

The yield over the two trials combined (Appendix S3, ‘Combined’ table) ranged from 8.159 ± 0.305 t/ha for edited line 178.75 to 6.026 ± 0.309 t/ha for TILLING 1AB. The yield of the combined edited lines was very slightly lower than their control, Cadenza, at 7.892 ± 0.279 t/ha compared with 7.982 ± 0.294 t/ha, a difference of 1.1% (*p =* 0.034). However, this could not be attributed to the editing process per se or to free asparagine levels (see below) because the yields of Lines 59.26 (8.086 ± 0.305 t/ha) and 59.84 (8.085 ± 0.305 t/ha), as with Line 178.35, were slightly higher than that of Cadenza, while the yields of Lines 23.60 (7.737 ± 0.309 t/ha) and 23.75 (7.393 ± 0.309 t/ha) were lower. Given that Lines 59.26 and 59.84 had never been studied in the field before, the fact that they did not show a reduction in yield is important, particularly in the context of their free asparagine levels (below). The TILLING lines, on the other hand, showed a significant (*p <* 0.001) reduction in yield of 22.5% compared with their control, Claire (6.266 ± 0.285 t/ha vs. 8.089 ± 0.292 t/ha).

TGW data for both trials are given in Appendix S4 with statistical analysis, and shown graphically in Figure 1, lower panel. Overall, TGW was higher in 2418 than 2318, despite the lower yields. Combining the data for the two trials (Appendix S4, ‘Combined’ table), Cadenza had the highest TGW at 52.16 ± 0.98 g, while TILLING ABD had the lowest (41.89 ± 1.23 g). The TGW of the combined edited lines was 45.21 ± 0.58 g, representing a significant (*p <* 0.001) reduction of 13% compared with Cadenza. The TGW and yield data together indicate that the edited lines produced more but smaller grains.

The TGW of the TILLING lines was also significantly (*p <* 0.001) lower than that of Claire (44.48 ± 0.98 g vs. 47.17 ± 0.98 g), a reduction of 5.7%. The fact that this decrease in TGW occurred in both the edited and TILLING lines suggests that it resulted from a reduction in free asparagine concentration. Consistent with this, the TGW of TILLING ABD (41.89 ± 1.23 g) was 8.5% lower (*p <* 0.001) than that of TILLING 1AB and 2AB combined (45.78 ± 0.88 g). However, there were no significant differences (*p =* 0.251) between the different edited lines (combined Lines 59.26 and 59.84 vs. combined Lines 23.60 and 23.75, and Line 178.35).

### 2.3 Total Nitrogen, Carbon and Sulphur

The total nitrogen (N) content of wheat grain is often used as an indicator of protein content. In the United Kingdom, for example, breadmaking wheat is expected to have a protein content of 13%, which equates to a total N content of approximately 2.3%, while soft biscuit wheats have a protein content below 10%, which equates to approximately 1.75% total N. Any effect of reducing free asparagine accumulation on total N content would therefore be very important. However, total N content showed little change in the edited lines compared with their control, Cadenza (Appendix S5). Comparing the combined edited lines with Cadenza across both trials there was a small (2%) but significant (*p <* 0.001) decrease in total N (2.44% ± 0.06% vs. 2.49% ± 0.06%). However, this resulted from a decrease in Line 178.35, with the other edited lines having very similar N content to Cadenza, and Line 23.75 having the highest total N of all the lines at 2.52% ± 0.06%. Furthermore, the combined TILLING lines had a significantly (*p <* 0.001) 6% higher N content than Claire (2.35% ± 0.05% vs. 2.21% ± 0.05%), with all of the Claire‐based lines having relatively high N content for a soft wheat. Overall, there was no evidence that interfering with asparagine synthesis reduced the total N content of the grain.

Total carbon (C) and sulphur (S) content were also analysed (Appendices S6 and S7). The differences in C content between the samples were very small, with all samples in the combined analysis ranging from 44.32% to 44.55% C. Total S showed some bigger differences (Appendix S7). The Cadenza‐based lines were higher in S than the Claire‐based lines (combined means of 1537 ± 7.02 parts per million (ppm) vs. 1452 ± 7.43 ppm) (*p <* 0.001). Lines 59.26 and 59.84 had the lowest S content of all the Cadenza‐based lines, with a combined mean of 1485 ± 8.86 ppm, 3.4% lower than Cadenza. Nevertheless, this was still higher than the mean for the Claire‐based lines. The S content of the TILLING lines was actually higher than that of Claire (combined mean of 1472 ± 8.0 ppm vs. 1392 ± 9.4 ppm) (*p <* 0.001).

### 2.4 Free Asparagine Concentration

**FIGURE 2:**
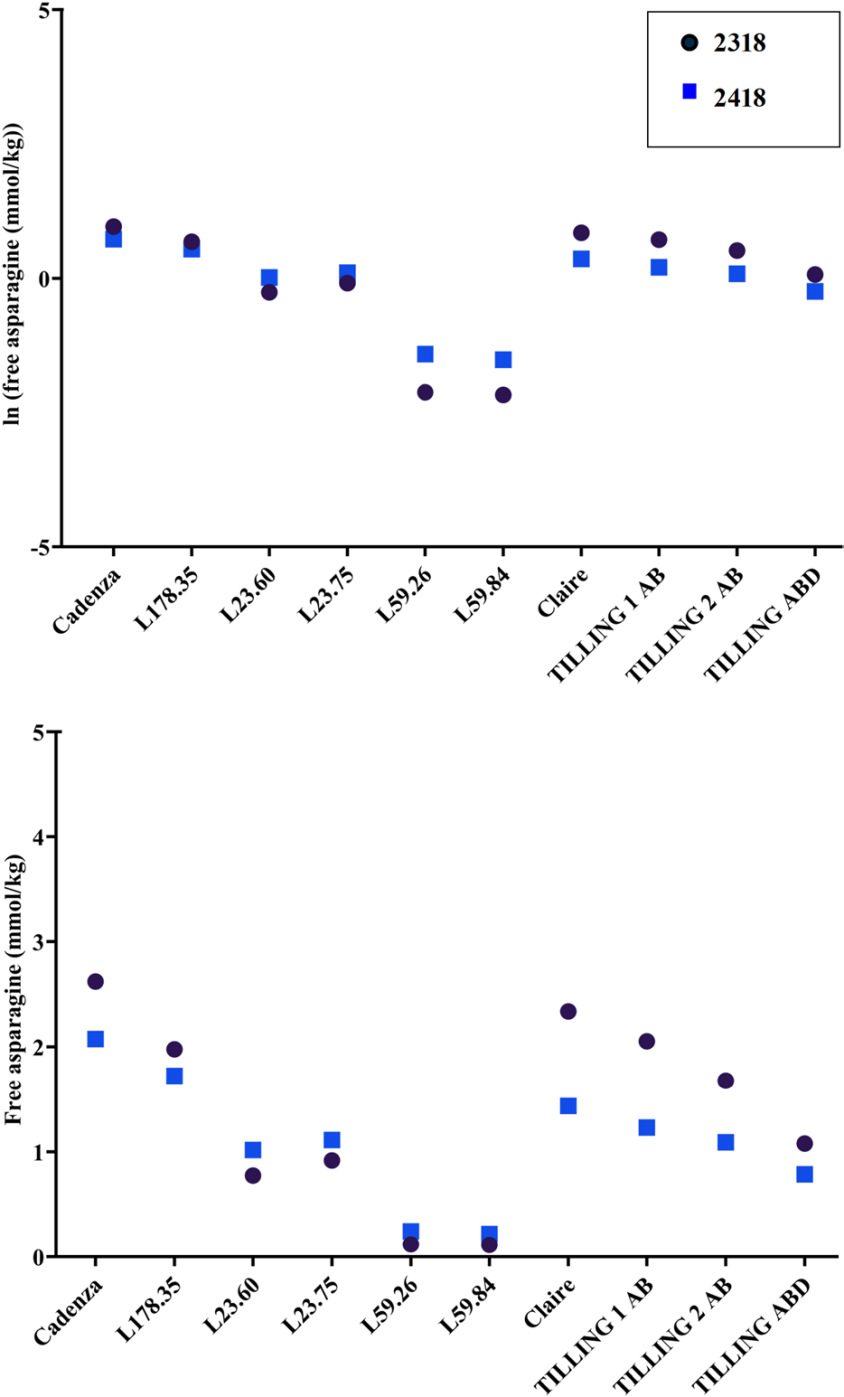
Free asparagine concentrations in the grain of wheat of different lines harvested from the 2318 and 2418 field trials as indicated, given on the natural log scale (upper panel) and as geometric (backtransformed) means (lower panel).

Grain was harvested and dried, and samples milled to fine, wholemeal flour before the concentrations of free amino acids were measured by HPLC. The full data sets for each trial are given in Appendices S8 and S9. Statistical analyses of the free asparagine data were performed after transformation to the log_e_ scale, with the geometric (backtransformed) means considered to be a better representation of the data than the arithmetic mean. The means and other statistical data are given in Appendix S10, with the log_e_ data for each trial shown graphically in Figure 2, upper panel, and the geometric means in Figure 2, lower panel.

Overall, free asparagine concentrations in both trials were higher than in the 2021–2022 field trial (Raffan et al. 2023), comparing the lines that were in all three trials. For Cadenza, for example, the mean concentrations of free asparagine in 2318 and 2418 were 2.62 and 2.08 mmol kg^−1^, respectively, compared with 1.06 mmol kg^−1^ in the 2021–22 field trial (Raffan et al. 2023). Free asparagine accumulation is very sensitive to environmental conditions (Lea et al. 2007), and the higher concentrations in 2318 and 2418 were probably caused by the difficult growing conditions, with both the 2022–2023 and 2023–2024 seasons having periods of both very wet and very dry weather (http://resources.rothamsted.ac.uk/environmental‐change‐network/yearly‐weather‐summaries#loaded). In 2023, for example, there were only 5.7 mm of rain in February, but March was very wet, with 117 mm of rain, adversely affecting the development of the crop emerging from winter that year. In 2024, on the other hand, February was extremely wet, with 130 mm of rain, and the wet conditions continued through to May, which saw 114 mm of rain.

A combined LMM meta‐analysis was used to analyse the free asparagine concentrations. Over the two trials combined, the mean free asparagine concentration for Cadenza was 2.33 mmol kg^−1^, while for Line 178.35 it was 1.85 mmol kg^−1^, for Lines 23.60 and 23.75 combined it was 0.95 mmol kg^−1^, and for Lines 59.26 and 59.82 combined it was 0.16 mmol kg^−1^. The difference between Cadenza and the edited lines was significant (*p <* 0.001), as were the differences between the different edited lines (*p <* 0.001). The concentration of free asparagine in Line 178.35 was 79% that of Cadenza, while in Lines 23.60 and 23.75 combined it was 41% and in Lines 59.26 and 59.84 combined it was an astonishing 7%.

The concentration of free asparagine in grain from Lines 23.60 and 23.75 was approximately 50% of that in Cadenza in the 2021–2022 field trial (Raffan et al. 2023), so the reduction in free asparagine in 2318 and 2418 was greater than in the earlier trial. In fact, free asparagine concentration increased in both 2318 and 2418 for all three genotypes compared with 2021–2022 but the concentration in Cadenza increased by more than that in the edited lines.

The TILLING lines also showed reductions in free asparagine concentration when compared with their control, Claire. Over the two trials combined, the mean concentration of free asparagine in the Claire samples was 1.83 mmol kg^−1^, whereas it was 1.47 mmol kg^−1^ in TILLING 1AB and 2AB combined, and 0.92 mmol kg^−1^ in the TILLING ABD line. The difference between Claire and the TILLING lines was significant (*p <* 0.001), as was the difference between the TILLING AB and ABD lines (*p <* 0.001). The concentration in the TILLING AB lines was 80% that of Claire, while that in the TILLING ABD line was 50% that of Claire.

### 2.5 Acrylamide in Bread and Biscuits

In order to show the effect of reducing free asparagine concentration in the grain on acrylamide formation in baked products, bread and biscuits were baked using flour prepared from a selection of grain samples from the 2318 trial. These were the Cadenza and Claire controls, CRISPR lines 23.60 and 59.26, and TILLING ABD, with grain from four plots being used for each line. These lines gave a range of high, medium and low free asparagine concentrations. The total protein content and Hagberg Falling Number (HFN) were measured in the five flour samples to check that they were suitable for baking. The total protein content was measured by the Bradford method and was 1.92 ± 0.04 g L^−1^ for Cadenza, and 2.00 ± 0.03 and 2.01 ± 0.26 g L^−1^ respectively for the edited lines, 23.60 and 59.26. The Claire and TILLING ABD lines were slightly lower at 1.93 ± 0.10 and 1.92 ± 0.08 g L^−1^, respectively, as would be expected with Claire being a biscuit wheat and Cadenza a bread wheat. There was no correlation between free asparagine concentration and total protein content.

The HFN is a measure of starch breakdown and, indirectly, α‐amylase activity. For bread, the ideal HFN range is generally considered to be 250–280 s. The HFN for Cadenza was within this range at 261 ± 11 s, but the others were higher: 388 ± 8 s for Line 23.60, 349 ± 21 s for Line 59.26, 322 ± 5 s for Claire and 312 ± 33 s for TILLING ABD.

**FIGURE 3:**
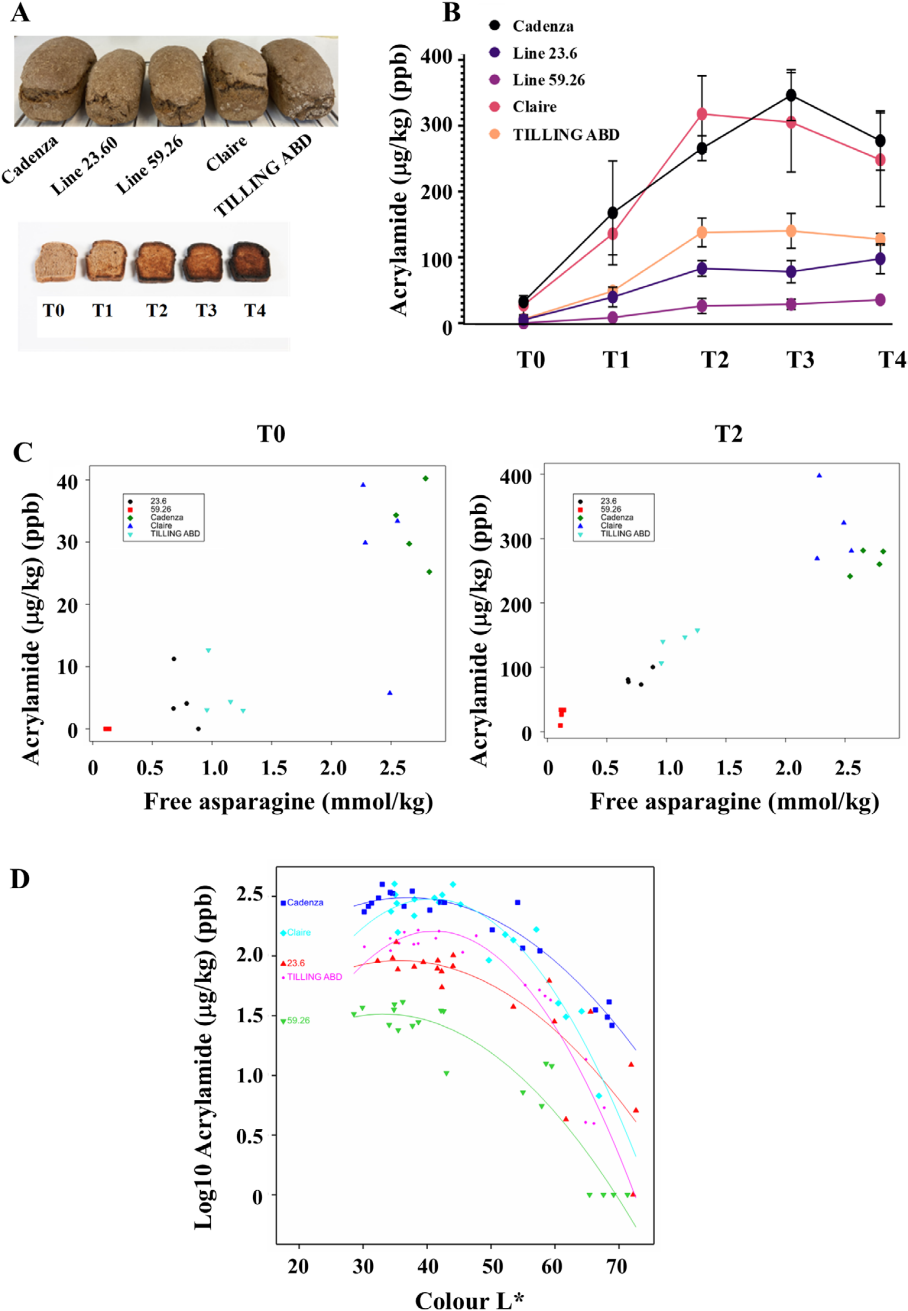
Acrylamide in bread and toast prepared from selected lines of wheat grown in field trial 2318. The lines were selected to provide a range of free asparagine concentrations. (A) Bread loaves and toast slices, with T0 untoasted bread, T1 toasted for 3 min, T2 for 3 min 30 s, T3 for 4 min and T4 for 4 min 30 s. (B) Acrylamide mean and standard error of the mean measured in toast prepared from the different lines, as indicated. (C) Acrylamide plotted against free asparagine concentration in T0 (left panel) and T2 (right panel) toast, as indicated, with data points for the different lines marked in different colours, as shown. (D) Acrylamide plotted against colour (L*) in toast, with separate quadratic curves for each line (*p* = 0.002).

Four loaves of bread were baked from each line, and typical loaves for each line are shown in Figure 3A. After baking, the height of each loaf was measured, with the mean for the Cadenza loaves being 7.38 ± 0.19 cm, compared with 6.78 ± 0.43 cm for 23.60, 6.93 ± 0.15 for 59.26, 7.43 ± 0.27 for Claire and 7.03 ± 0.22 for TILLING ABD. There was, therefore, some evidence of smaller loaves being produced from the edited lines, which would tally with the higher HFN for those lines; however, the differences were not significant (*p* > 0.05).

For each loaf, five slices were cut and four of these slices were toasted for 3 min (T1), 3 min 30 s (T2), 4 min (T3) and 4 min 30 s (T4), the untoasted slice being designated T0. The appearance of typical slices is shown in Figure 3A, and colour values (L* [dark to light axis], a* [red to green axis] and b* [yellow to blue axis]) are given in Appendix S11.

Acrylamide was measured in the slices after grinding the whole slice to a fine powder. The data and statistical analysis are given in Appendix S12, with the means and standard deviations for the different lines shown graphically in Figure 3B. The two edited lines and the TILLING line showed dramatic and significant reductions in acrylamide formation compared with their controls, with Line 59.26 significantly lower than Line 23.60 (*p <* 0.001 for Cadenza vs. Line 23.60; *p <* 0.001 for Line 23.60 vs. Line 59.26; *p <* 0.001 for TILLING ABD vs. Claire). Indeed, acrylamide in untoasted bread (T0) from Line 59.26 was below detection levels, compared with 32.38 μg kg^−1^ in bread made from the Cadenza control. The concentration of acrylamide in bread made from Line 23.60 flour was 4.65 μg kg^−1^, only 14% that for Cadenza, while the concentration in bread from the TILLING ABD line was 5.75 μg kg^−1^, only 21% that for Claire (27.04 μg kg^−1^).

The concentration of acrylamide rose sharply in bread from all of the lines when toasted, with the highest concentrations seen in the T3 toast from Cadenza (346.32 μg kg^−1^) and Claire (305.19 μg kg^−1^). Acrylamide reduced with longer toasting for these breads, to 277.24 μg kg^−1^ in T4 toast for Cadenza and 248.25 μg kg^−1^ for Claire. A similar reduction after extended cooking periods has been reported for simple wheat, rye and potato cakes, as free asparagine and reducing sugars become depleted and acrylamide breaks down (Elmore et al. 2005). However, the T4 toast for all lines was starting to look burned (Figure 3A) and would almost certainly be unpalatable and rejected by consumers. In T3 toast made from Line 59 bread, the concentration of acrylamide was 28.38 μg kg^−1^, only 8% that of Cadenza, while the concentration for Line 23.60 was 78.07 μg kg^−1^, 23% that of Cadenza. The concentration in TILLING ABD T3 toast was 140.06 μg kg^−1^, 46% that for Claire.

There were significant (*p* < 0.001) correlations between free asparagine concentration and acrylamide formation, and the data (Appendix S13) are shown graphically for bread (T0) and toast (T2) in Figure 3C. In fact, the correlation became tighter (less variable) when the bread was toasted. There was also a strong relationship between acrylamide formation and colour L*, with higher acrylamide associated with darker toast colour (Figure 3D; Appendix S14) (Pearson's correlation coefficient [*R*] for the overall relationship between colour (L*, log scale) and acrylamide was −0.6129). This is to be expected because the compounds that cause the browning of baked and toasted foods are also products of the Maillard reaction. Importantly, however, the relationship between colour and acrylamide formation was altered in the edited and TILLING lines, with more colour forming for less acrylamide, resulting in the data being best represented by separate quadratic curves for each line (*p* = 0.002) (Figure 3D).

**FIGURE 4:**
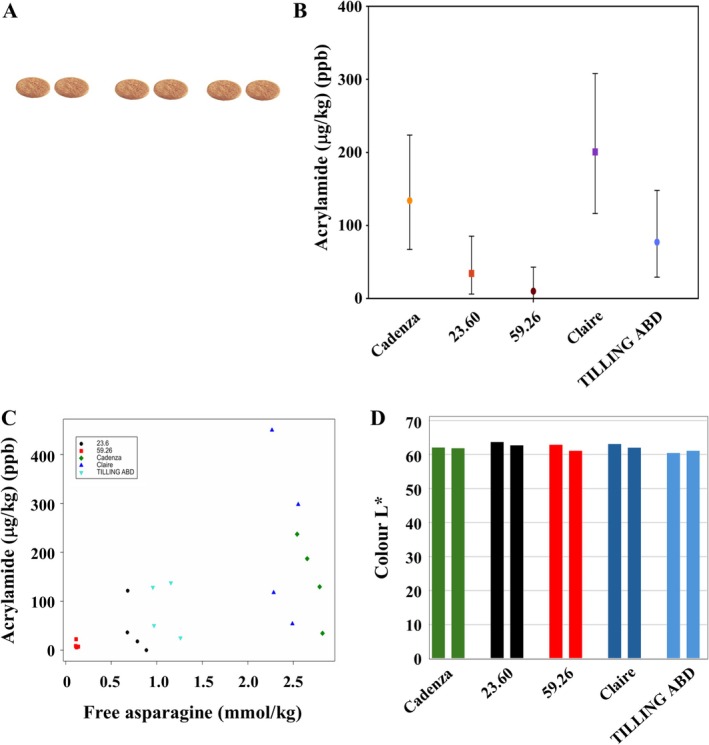
Acrylamide in biscuits prepared from selected lines of wheat grown in field trial 2318. The lines were selected to provide a range of free asparagine concentrations. (A) Representative biscuits. (B) Acrylamide (geometric mean and 95% confidence levels) measured in biscuits prepared from the different lines, as indicated. (C) Acrylamide plotted against free asparagine concentration in biscuits, with data points for the different lines marked in different colours, as shown. (D) Colour (L*) of biscuits prepared from the different lines, as indicated, with the left bar for each line representing upper surface colour and the right bar bottom surface colour.

Biscuits are considered to be of relatively high risk for acrylamide content, with a current EU Benchmark Level of 350 μg kg^−1^ (parts per billion [ppb]). Biscuits were prepared from the same wholemeal flour samples used in the bread experiment, with water, sugar, sunflower oil and baking powder added according to a standard recipe (Musa et al. 2024). Four batches were baked for each line, and representative biscuits are shown in Figure 4A.

Acrylamide was measured in the biscuits after grinding to a fine powder, and the results and statistical analysis are given in Appendix S15, with the geometric means and 95% confidence levels for the different lines shown graphically in Figure 4B. As with the bread and toast, the two edited lines and the TILLING line showed dramatic reductions in acrylamide formation compared with their controls, with the concentration of acrylamide in the biscuits from Line 59.26 at 10.1 μg kg^−1^ (geometric mean) compared with 134 μg kg^−1^ for Cadenza, representing a 92% reduction. The concentration of acrylamide in biscuits made from Line 23.60 was also much lower than for Cadenza, at 34.4 μg kg^−1^, a 74% reduction. The difference between the edited lines and Cadenza was significant (*p* = 0.003). Biscuits made from Claire flour had the highest acrylamide concentration at 201 μg kg^−1^, although there was considerable scatter in the data (Figure 4B). In comparison, the acrylamide concentration in the biscuits made from the TILLING ABD line was 77.3 μg kg^−1^, representing a 62% reduction. The difference between the two lines was significant (*p =* 0.031).

As with the toast, the relationship between free asparagine concentration and acrylamide formation was significant (*p* = 0.0072), and the data (Appendix S16) are shown graphically for the different lines in Figure 4C. In contrast to acrylamide, biscuit colour was almost unchanged between the different lines (Figure 4D; Appendix S17), showing, as with the toast experiment, that the relationship between colour and acrylamide formation was altered in the edited and TILLING lines, with similar colour forming for less acrylamide.

## 3 Discussion

Here, we report the results of 2 years of field trials of wheat genotypes produced using CRISPR/Cas9 (the edited lines, Cadenza background) and chemical mutagenesis (the TILLING lines, Claire background). The CRISPR/Cas9 editing was designed to target the asparagine synthetase‐2 (*TaASN2*) genes (Raffan et al. 2021), and A genome (*TaASN‐A2*) (Line 178.35) and total knockouts of *TaASN2* (Lines 23.60 and 23.75) were included in the trial. Another total *TaASN2* knockout, Line 59, was found to have additional edits in the A and B genome versions of the closely related gene, *TaASN1*. This line had never been grown in field trials before. The trials also included for the first time a total null TILLING line for *TaASN2* (TILLING ABD).

*TaASN2* is expressed seed‐specifically, with highest expression in the embryo during mid‐development, whereas *TaASN1* is expressed more widely in the plant (Gao et al. 2016; Curtis et al. 2019). It is possible that a full knockout of *TaASN1* would have deleterious effects on plant development, but this requires further investigation. Non‐Triticeae cereals, such as maize ( *Zea mays* ) and rice ( *Oryza sativa* ), do not have an equivalent gene (Raffan and Halford 2021). Rice lines lacking a functional *OsASN1* gene (actually equivalent to *TaASN4*) showed effects on plant height, root length and tiller number (Luo et al. 2019), but rice has only two asparagine synthetase genes, the five‐member gene family being unique to the Triticeae tribe (Raffan and Halford 2021), so the two species are not directly comparable.

The seed‐specific expression of *TaASN2* made it an obvious candidate for genetic interventions to reduce free asparagine accumulation in the grain. The resulting almost 60% reduction in free asparagine concentration in the *TaASN2* total knockout lines somewhat validated this strategy. However, free asparagine still accumulated to 41% of that in the control, and a further big reduction was brought about by the partial knockout of *TaASN1*. *TaASN1* is also expressed in the grain, although at much lower levels than *TaASN2*, but it is also expressed in roots and, to a lesser extent, in stems. It is possible that post‐transcriptional regulation means that *TaASN1* gives rise to disproportionately more protein than *TaASN2*. It is also possible that the TaASN1 enzyme is more active than TaASN2, although we did not see evidence of this in heterologously‐expressed enzymes (Xu et al. 2018). The third and perhaps most plausible explanation is that a considerable proportion of the free asparagine that accumulates in the grain is imported from elsewhere in the plant and that less is available for import if *TaASN1* expression is reduced. Consistent with this, over‐expression of asparagine synthetase in maize under the control of a rice actin (*ACT1*) gene promoter resulted in higher asparagine accumulation in leaf tissue and higher kernel protein levels (Crowley et al. 2025). The authors of that study attributed higher asparagine seed tissue to transport of asparagine from the leaf to the seed. Given this context, it is important that the asparagine synthetase knockouts in our study did not show a reduction in total seed nitrogen, which is generally accepted as an indicator of seed protein content.

Asparagine synthetase has also been linked with seed protein content in teosinte ( *Z. mays* ssp. *parviglumis* ) (Huang et al. 2022), being encoded by a major high‐protein quantitative trait locus, Teosinte High Protein 9 (*THP9*). Over‐expression of *THP9* in a maize inbred line carrying a deletion in the corresponding native gene increased seed protein content and nitrogen use efficiency, as did introgression of the gene into maize inbreds and hybrids (Huang et al. 2022). However, not surprisingly, it also led to a rise in free asparagine concentration throughout the plant.

These studies have not yet led to the development of commercial GM maize varieties. Maize may be predominantly used for animal feed and that would presumably be the target market for high‐protein varieties, but maize‐based snacks, flatbreads, pancakes, tortillas, tamales and other products are staples in the human diet in many countries, notably in South America. In our view, therefore, the development of high asparagine varieties would be irresponsible, regardless of potential improvements in kernel protein content or nitrogen use efficiency.

Over‐expression of asparagine synthetase in maize did not affect yield, and we also observed no consistent effect on yield in our knockout lines. The fact that free asparagine concentration in the grain can be reduced by over 90%, as it was in Lines 59.26 and 59.84, shows that wheat accumulates far more free asparagine in the grain than it needs for synthesising proteins. This is consistent with the hypothesis that free asparagine is used as a rapidly mobilisable nitrogen store in cereal grains, a hypothesis that was first put forward by Shewry et al. (1983). Yield of the TILLING lines, on the other hand, was reduced by almost a quarter compared with Claire, and a similar reduction was observed in an earlier field trial of partial TILLING knockouts (Raffan et al. 2023). The TILLING lines will carry many more mutations than those identified in the *TaASN2* genes, of course, and it is likely that this genetic baggage was responsible for the yield penalty in those lines, rather than the reductions in free asparagine concentration. It is, therefore, reasonable to expect that the yield penalty could eventually disappear if the *TaASN2* mutations were introgressed into wheat breeding lines. Nevertheless, the contrast with the edited lines was stark, and highlighted the advantages of targeted as opposed to random mutagenesis.

Yield was maintained in the edited lines despite a reduction in thousand grain weight (TGW) compared with the Cadenza control, indicating that the edited lines produced more but smaller grains. It is possible that the lower TGW was caused by the changes in free asparagine content, but the effect was inconsistent and requires further investigation. In the 2418 trial, for example, Line 178.35 had the lowest TGW, despite the 23 and 59 lines having much lower concentrations of free asparagine.

The primary aim of the project, to demonstrate that a reduction of free asparagine in the grain of wheat could be achieved and maintained in the field, without affecting yield, was achieved. Indeed, the reduction of over 90% in Lines 59.26 and 59.84 was astonishing, with impressive reductions in the *TaASN2* knockouts produced by both editing and TILLING as well (59% and 50%, respectively). These reductions compare with a decrease of 28% seen previously in A genome TILLING nulls in the Cadenza background, and 24%–34% in the tetraploid Kronos background (Alarcón‐Reverte et al. 2022).

Free asparagine concentration is affected by environmental and crop management as well as genetic factors, and that caveat must be considered when comparing free asparagine concentrations across different studies. However, the concentration of 0.16 mmol kg^−1^ observed in the Lines 59.25 and 59.84 combined in this study is far lower than anything reported for currently available varieties. Curtis et al. (2018), for example, reported concentrations of 1.521–2.687 mmol kg^−1^ in 25 UK varieties grown in 2011–2012, and 0.708–11.29 mmol kg^−1^ in 59 varieties grown in 2012–2013, while Žilić et al. (2017) reported that wheat in Serbia typically had concentrations of free asparagine in the grain between 3.0 and 4.5 mmol kg^−1^, with an exceptionally low variety (Zemunska rosa) having a concentration of 1.45 mmol kg^−1^. An Italian study of wheat grown in field trials at three locations over 2 years reported free asparagine concentrations of between 0.99 and 2.82 mmol kg^−1^ in year 1 and between 0.55 and 2.84 mmol kg^−1^ in year 2 (Tafuri et al. 2023). We conclude that the editing approach enabled us to produce wheat with free asparagine concentrations not only substantially lower than that in the Cadenza control but also well below the normal range for wheat, and consistently so.

Evidence to date suggests that conventional breeding would be unlikely to deliver a similar improvement. Several studies have identified quantitative trait loci (QTL) for grain asparagine content (Emebiri 2014; Peng et al. 2018; Rapp et al. 2018; Oddy, Chhetry, et al. 2023; Tafuri et al. 2025), but the search for strong QTL that are consistent across multiple studies has so far been frustrating (reviewed by Kaur and Halford 2023).

The reduction in free asparagine produced a concomitant reduction in acrylamide formation in bread, including after toasting, and biscuits, with strong correlations between free asparagine concentration and acrylamide formation in both food types. The formation of acrylamide in wheat products depends upon many factors, from the effects of variety selection and crop management on flour composition through to product recipes, measures taken by processors, and the actions of food preparers in the catering industry and consumers in the home (reviewed by Kaur and Halford 2023). The EU's regulations on acrylamide (currently Regulation [EU] 2017/2158) put all the responsibility for the presence of acrylamide in food products on food businesses, rather than their supply chain. It is even the responsibility of food businesses to source low asparagine grains rather than the supply chain to provide them. To date, breeders have generally resisted pressure from further up the supply chain to develop low asparagine wheat varieties. However, the pressure is likely to increase if the EU reduces the Benchmark Levels for acrylamide and introduces Maximum Levels, and the EU's regulations affect not only its Member States but also its many trading partners.

The commercial success of low asparagine wheat would, of course, require that it could be processed into products that were acceptable to food businesses and their consumers. Our baking studies were small‐scale, but the only potential problem that we identified was a relatively high Hagberg Falling Number (HFN), which may have affected the volume of the bread loaves produced in the study. Bread producers are already familiar with this problem and its solution, which is to add α‐amylase to the dough before proofing. A high HFN is also associated with resistance to pre‐harvest sprouting (see Hull et al. 2024 for recent review), and that aspect of the low asparagine lines requires further investigation.

In conclusion, we have shown that large reductions in free asparagine concentration in wheat grain can be achieved using both CRISPR and chemical mutagenesis (TILLING). CRISPR has clear advantages over TILLING, notably in this case in that no yield drag was evident in the CRISPR lines while the yield of the TILLING lines was substantially reduced. Stacking TILLING mutations would also become very complicated and time‐consuming with a more complex gene family. However, new varieties produced using CRISPR still face regulatory barriers in many countries, while varieties produced using TILLING do not; a situation that has no scientific basis. This study has coincided with the adoption of new regulations for genome edited crops in England, which will class transgene‐free genome edited plants as precision bred organisms (PBOs) (the Genetic Technology [Precision Breeding] Act of March 2023; https://bills.parliament.uk/bills/3167). The system of approving PBOs for marketing under this legislation opened in November 2025 and must be simple to navigate if breeders are to adopt the technology.

This is important because the availability of low acrylamide wheat could enable food businesses to comply with evolving regulations on acrylamide without costly changes to production lines or reductions in product quality. It could also have a substantial impact on dietary acrylamide intake for consumers.

## 4 Experimental Procedures

### 4.1 Field Trials and Grain Analyses

Field trials were carried out on Pastures Field, Rothamsted Research, Harpenden; Grid reference TL122134. The 2022–2023 trial, reference 2318, was drilled on the 14th November 2022 and harvested on 25th August 2023. The relatively late sowing was caused by wet weather in the preceding weeks and the late harvest by a relatively cold and wet spring. The 2023–2024 trial, referenced 2418, was drilled on 7th November 2023, again delayed by wet weather, and harvested on 28th August 2024. The layout of each trial is shown in Appendix S2. The total size of each trial was 2258 m^2^ (67 m × 33.7 m), including a 6 m pollen barrier. There were 56 × (6 m × 1.8 m) plots, including five of each of edited lines 178.35, 23.60, 23.75, 59.26 and 59.84, as well as TILLING lines 1AB, 2AB and ABD. There were eight plots of each of the Cadenza and Claire controls. In each trial, lines were allocated in randomised fashion to plots in a 7 × 4 row‐column design within each of two blocks of 28 plots.

After harvest, a sub‐sample of grain from each plot was weighed to calculate fresh weight. This sub‐sample was then oven‐dried overnight at 105°C and then re‐weighed to measure dry weight. The percentage reduction in weight from lost moisture content was then used to adjust yield estimates taken at harvest on the combine and to calculate grain yield at 85% dry matter. Thousand grain weight (TGW) measurements were subsequently taken by counting 500 seeds using a seed counter (model C1; Elmor AG, Schwyz, Switzerland), drying overnight at 105°C, and then weighing.

Hagberg falling number (HFN) was analysed using a Perten falling number 1000 (PerkinElmer, Shelton, CT, USA). The required sample weight was calculated depending on the moisture of the flour. Then, 25 mL water were added and mixed with flour until no dry sample pockets were present, at which point the tube was ready for testing. Each measurement was made in duplicate.

### 4.2 Total Nitrogen (N), Carbon (C) and Sulphur (S)

These analyses were performed at the Analytical Chemistry Facility at Rothamsted Research. Total N and C were measured by combustion analysis (Dumas digestion method) using a LECO CN628 Combustion Analyser (LECO, Stockport, UK). S concentration was measured using an Agilent 5900 SVDV Inductively Coupled Plasma—Optical Emission Spectrometer (ICP‐OES) (Agilent Technologies LDA UK Limited, Stockport, UK). Flour for the S analysis was prepared by microwave digestion (MARS; CEM Corporation, Matthews, NC, USA) using nitric acid in closed vessels.

### 4.3 Free Amino Acid Analyses

Free amino acids were extracted from wholemeal flour samples with hydrochloric acid and derivatised with *o*‐phthalaldehyde. Concentrations of free amino acids were then measured by Curtis Analytics (Harpenden, UK), using high‐performance liquid chromatography with fluorescence detection, as described previously (Raffan et al. 2021).

### 4.4 Preparation of Biscuits, Bread and Toast

Sugar, sunflower oil, baking powder, yeast and sodium chloride were purchased at a local supermarket in Harpenden, UK. Wheat grain samples from five selected lines, Cadenza (control), 59.26, 23.60, Claire (control) and Tilling ABD, were milled into wholemeal flour using an ultra‐centrifugal mill (ZM 200; Retsch, Düsseldorf, Germany). In each case, grain was analysed from four different plots, amounting to a total of 20 samples. After milling, the samples were stored in a cool, dry place. Moisture content was determined using a Bruker minispec mq‐20 NMR analyser (Bruker Corporation, Billerica, MA, USA).

Four loaves of bread were baked from each line. Bread dough was prepared according to Fredriksson et al. (2004). Flour, dry yeast, tap water and salt were mixed, and the dough was kneaded and left to rise at 35°C (±2°C) for 30 min at 55% relative humidity. The dough was kneaded again and then placed into bread tins and left to rest for 5 min before transferring to the oven, arranged according to a one‐way design (randomised blocks). It was baked at 230°C for 30 min, after which the height of each loaf was measured. The bread was then cooled at room temperature for 1 h and stored for further analysis.

For each loaf, five slices were cut using an electric knife (Cookworks, UK). One slice (T0) was not toasted, while the other four were toasted using a home toaster (Tesco Stores Ltd., Welwyn Garden City, UK), with T1, T2, T3 and T4 toasted for 3 min, 3 min 30 s, 4 min and 4 min 30 s, respectively. The sample weight loss was recorded for each slice by calculating the difference in weight (%) for each slice before and after toasting.

Biscuits were made in four batches for each line. Biscuit dough was prepared according to Musa et al. (2024) with slight modifications, with water added to the wholemeal flour to 48%, sugar to 14%, sunflower oil to 12.2% and baking powder (sodium bicarbornate) to 0.33%. The dry ingredients were mixed at low speed for 30 s using a hand mixer (Aenader, UK) and then the water and oil were added and mixed for 3 min at medium speed and 30 s at high speed. The dough was then sheeted at a thickness of 0.5 cm and baked at 230°C for 20 min, again arranged in the oven according to a one‐way design in randomised blocks. After baking, biscuits were cooled at room temperature for 30 min and stored in zip‐lock bags for further analysis.

### 4.5 Colour Analysis

For biscuits and slices of bread, toasted and untoasted, the colour was measured using a Colour Muse (Variable Inc., Chattanooga, TN, USA). Colour values L*a*b* were obtained for the bottom and top surface of the biscuits and both sides of the bread/toast slices.

### 4.6 Acrylamide Analysis

Sample preparation and extraction were done according to Halford et al. (2012) with slight modifications. Bread and biscuit samples were milled to a fine powder using a coffee grinder (G100; Kenwood, Solihull, UK). The ground sample (0.25 g) was transferred to a 15‐mL Falcon tube and 0.5 mL of ^13^C_3_‐acrylamide internal standard (100 μg/L in water) and 9.5 mL water were added. Samples were shaken vigorously for 20 min at 1800 rpm and centrifuged at 15°C for 10 min at 8422 *g*. An aliquot from the aqueous layer (2 mL) was removed and filtered with a 0.2‐μm syringe filter. The filtered samples were transferred to 2‐mL amber vials with snap caps and placed in an autosampler tray at 4°C for analysis. Samples were analysed by liquid chromatography–tandem mass spectrometry (LC–MS/MS), using an Agilent 1200 HPLC with 6410 triple‐quadrupole mass spectrometer. Ionisation was achieved with an electrospray ion source operating in positive ion mode. The mobile phase was water:methanol (9:1) containing 0.1% formic acid at a flow rate of 0.4 mL/min. An isocratic separation was carried out at 60°C using a 150 × 3.0 mm Hypercarb column with a 10 × 3.0 mm Hypercarb precolumn (both 5 μm particle size; Thermo Fisher, Waltham, MA, USA). Injection volume was 25 μL. The transitions *m/z* 72 → 55 and 72 → 27 were measured for acrylamide, and the transition *m/z* 75 → 58 was measured for ^13^C_3_‐acrylamide.

### 4.7 Statistical Analyses

Statistical analyses were performed using GenStat (VSN International Ltd., Hemel Hempstead, UK) (VSN International 2022, 2023).

## Funding

This work was supported by Biotechnology and Biological Sciences Research Council (BB/T017007/1, BB/W007134/1).

## Conflicts of Interest

The authors declare no conflicts of interest.

## Supporting information


**Appendix S1:** CRISPR/Cas9 editing in *TaASN1* genes of Line 59.
**Appendix S2:** Trial layouts for 2318 and 2418.
**Appendix S3:** Yield of grain from field trials 2318 and 2418, with statistical analysis.
**Appendix S4:** Thousand grain weight (TGW) of grain from field trials 2318 and 2418, with statistical analysis.
**Appendix S5:** Total nitrogen (N) (%) in grain from field trials 2318 and 2418, with statistical analysis.
**Appendix S6:** Total carbon (C) (%) in grain from field trials 2318 and 2418, with statistical analysis.
**Appendix S7:** Total sulphur (S) (parts per million [ppm]) in grain from field trials 2318 and 2418, with statistical analysis.
**Appendix S8:** Free amino acid concentrations (mmol/kg) in grain harvested from the 2318 field trial.
**Appendix S9:** Free amino acid concentrations (mmol/kg) in grain harvested from the 2418 field trial.
**Appendix S10:** Statistical analysis of free asparagine concentrations (mmol/kg) from the 2318 and 2418 field trials.
**Appendix S11:** Bread/toast colour L*a*b*.
**Appendix S12:** Acrylamide formed in bread and toast, with statistical analysis.
**Appendix S13:** Free asparagine concentration versus acrylamide in T0 and T2 bread/toast.
**Appendix S14:** Bread and toast acrylamide versus colour L*.
**Appendix S15:** Acrylamide formed in biscuits, with statistical analysis.
**Appendix S16:** Free asparagine concentration versus acrylamide in biscuits.
**Appendix S17:** Biscuit colour L*a*b*.

## Data Availability

The data that support the findings of this study are available in the Supporting Information.
